# Usability and Acceptability of a Mobile App to Help Emerging Adults Address their Friends' Substance Use (Harbor): Quantitative Study

**DOI:** 10.2196/16632

**Published:** 2020-11-05

**Authors:** Kyle Michael Bennett, Kelly Lynn Clary, Douglas Cary Smith, Carol Ann Lee

**Affiliations:** 1 University of Illinois Urbana-Champaign Urbana, IL United States; 2 Texas State University San Marcos, TX United States

**Keywords:** young adults, substance abuse, peer influence, mobile applications, mobile phone, community-based participatory research, crowdsourcing

## Abstract

**Background:**

Technology-assisted intervention and prevention strategies present opportunities for substance use–related research with emerging adults (EAs) and their peers. Emerging adulthood is a developmentally distinct period in which individuals between the ages of 18 and 29 years undergo unique emotional, cultural, developmental, and biological changes as they transition into adulthood. Crowdsourcing, or gathering feedback from a large group within web-based communities, offers researchers a unique and cost-effective way to obtain large amounts of information in a short period.

**Objective:**

This paper presents market feedback obtained via Amazon’s Mechanical Turk from EAs (N=458) on the acceptability and utility of brief intervention scripts for a smartphone app currently under development. The mobile app, Harbor, teaches friends of EAs with substance use problems effective and supportive strategies for helping their friend make changes in their substance use behavior.

**Methods:**

We examined feedback on the wording of the intervention scripts and estimated the market size of EAs who may use this app. Furthermore, we calculated correlations between script ratings and measures of personal risky drinking (ie, Alcohol Use Disorder Identification Test) and the participants’ use of confrontational, enabling, or supportive behaviors with an existing friend.

**Results:**

Approximately half of our sample (208/458, 45.4%) indicated that they had a close friend for whom they had concerns about their substance use, suggesting a potentially high demand for an app such as Harbor. Initial findings suggest that peers who engage in less enabling behaviors with friends who have a substance use problem exhibited lower risky drinking behaviors overall (*r*_206_=−0.501; *P*<.001). Concerning acceptability, 98.0% (449/458) of the sample rated the scripts’ dialogue as either somewhat, moderately, or extremely realistic (mean 3.92, SD 0.48) on 5-point Likert scale items. Finally, 95.4% (437/454) of respondents indicated that the scripts would be at least slightly helpful for training peers to help their friends with substance use issues. Finally, individuals who were better able to identify enabling language in enabling scripts self-reported fewer enabling behaviors toward their friend’s substance use (*r*_206_=−0.236; *P*=.001).

**Conclusions:**

There exists a demonstrated level of desirability and acceptability among EAs for a mobile app such as Harbor. EAs who wish to engage in more supportive behaviors with their friends who engage in substance use and who are amenable to assisting their friends with sobriety likely would use and benefit from this app.

## Introduction

### Background

National surveys show that emerging adults (EAs; 18-29 years) in the United States have higher rates of substance use than any other age group [1]. To make matters worse, EAs also have inferior treatment engagement rates compared with other age groups [1]. In a nationally representative sample of EAs (N=19,312), only 11% (n=2124) of those diagnosed as having a substance use disorder received related treatment [2]. Given this treatment gap, it is important to think about concerned significant others (CSOs), such as peers, of EA substance users as potential first responders. The reach of social networks in the United States peaks during the emerging adulthood years, which points toward the importance of close friends and the influence they can have during this time [3]. This paper presents data from a crowdsourcing study to inform the development of a mobile app that harnesses the social support of such peers for EA substance users.

Online survey research (ie, crowdsourcing) allows investigators a flexible and convenient means of collecting data on a wide variety of issues and topics. Crowdsourcing is the practice of collecting information or feedback on a project or task by enlisting the assistance of large samples of people, typically via the internet. In addition to the relative ease with which researchers can collect online data, especially with EAs, crowdsourcing is as reliable as or even more reliable than traditional EA sampling methods [4]. Crowdsourcing respondents tend to be more ethnically diverse [4], which is particularly important in the development of substance use interventions. That is, individuals in clinical trials are less ethnically diverse, less clinically impaired, more highly educated, and more frequently employed than individuals receiving treatment in community settings [5]. In short, online crowdsourcing may assist intervention developers in acquiring more diverse samples of EAs.

Following the principles of community-based participatory research (CBPR) in the development of the Harbor mobile app (eg, collaborating with community members and translating knowledge gained through the partnership into specific actions), we conducted a crowdsourcing study with a large sample of EAs (N=458). Combining aspects of CBPR with this crowdsourcing project may encourage a sense of empowerment and collective ownership in EA substance use research [6]. Participants gave feedback on whether they considered themselves to have a friend experiencing substance use issues and how they viewed the intervention scripts for Harbor. In addition, among those with friends with substance use problems, we analyzed associations between their intervention script feedback and measures of actual interactions they had with friends. Finally, we analyzed whether and how participants’ personal alcohol use was associated with script ratings.

Harbor helps EAs (aged 18-29 years) assist their friends in making changes to their substance use behaviors. It is an adaptation of a family therapy model called the Community Reinforcement Approach With Family Training (CRAFT), where CSOs learn communication skills to help individuals who demonstrated risky or problematic substance use behaviors [7].

### Objectives

Specifically, we sought to answer the following 3 questions:

1. What is the potential market size of EAs who may use the Harbor mobile app?
2. What do potential Harbor users think of our intervention screenshots/scripts?
3. How do screenshot/script ratings associate with self-reported participant behaviors in response to existing friends experiencing substance use problems?

Regarding the hypotheses, we first proposed that a large proportion of EAs would have friends with substance use problems. Second, based on prior acceptability studies for our face-to-face model, we hypothesized that participants would rate the scripts favorably. Third, we hypothesized that intervention script ratings would correlate negatively with actual self-reported interactions with their friends. Specifically, more confrontational or enabling friends would view the supportive intervention script (ie, CRAFT procedures script) as less helpful. Similarly, confrontational or enabling friends would rate the intervention scripts as less confrontational or enabling, respectively. We based hypotheses on the logic that such friends possess biases toward how they currently interact with their EA friends who misuse substances. Finally, we hypothesized that participants with riskier alcohol use would rate scripts as less enabling and self-report more frequent enabling behaviors toward their friends’ substance use compared with individuals with less risky alcohol use.

## Methods

### CBPR

CBPR promotes participant involvement and feedback in all stages of the research process and has resulted in greater inclusion of users’ experiences in behavioral health intervention development processes overall [8-11]. As part of this effort, there has been an increased focus on the expansion of patient-centered medicine [12], shared decision-making practices [13], and patient-friendly forms of communicative implementation as a whole [14]. Central to the CBPR ideology is the notion that by embracing and incorporating the experiences of end users, researchers reduce the risk of misunderstanding individuals’ health-seeking behaviors, which ultimately lowers the risk of undermining clinical practice [15]. Cognizant of and responsive to unique cultural, social, and economic factors affecting EA communities, CBPR is a move toward reconciliation, reciprocity, and production of culturally relevant prevention measures for this population. In this regard, a CBPR approach is well suited for promoting the utilization of substance use treatment among EAs.

Integrating end user feedback during the initial development may help improve the effect sizes of technological interventions, which typically have small effect sizes [16,17]. Thus, lay knowledge and expertise may help improve intervention outcomes in the field [18]. In addition, sustaining intervention or prevention strategies with a target population proves difficult [19], but seeking input from community members in research design and achieving shared health outcomes increases program sustainability [20]. This study asked EAs for initial feedback on script wording and the app’s quality to drive revisions. Individuals with lived experience can later review these revisions.

### Harbor: Background and Intervention Philosophy

In 2 prior studies, the authors adapted the CRAFT model for use with peer dyads [21,22]. At its core, CRAFT is a behavioral family therapy model for the CSOs of individuals with substance use disorders [7], and most research on CRAFT targets spouses. The CRAFT model emphasizes that CSOs attempt to interrupt their loved ones’ substance use by (1) arranging prosocial activities at times when they would likely use, (2) refraining from enabling their substance use by not doing pleasant things with them while they are using or doing things to prevent the natural consequences of their use, and (3) avoiding confrontation while their significant other is intoxicated. Essentially, CRAFT trains CSOs to reward substance-free behaviors and not reward substance-using behaviors. The overarching idea is that individuals will choose a substance-free life if it is more rewarding than one involving substances.

Previous research indicates that EAs are amenable to helping their close friends by learning the behaviors associated with the CRAFT model. However, they were less likely to want to disassociate with friends when their friends were using [21]. Thus, the peer dyad version of CRAFT, called the Peer-Enhanced Community Reinforcement Approach (Peer-CRA), de-emphasized this feature, which appears more suitable for those in romantic and cohabitating relationships. In addition, in a preliminary study, both peers and EAs with substance use problems who received Peer-CRA significantly reduced their substance use [22]. Despite promising findings, one issue in a previous face-to-face study was that most peers only attended 1 session and did not receive the full CRAFT model as planned. Ideally, the development of a mobile app will aid in the delivery of this content, supporting peer first responders whose EA friends may have not yet received any sort of substance use intervention and/or treatment.

### Harbor App Design and Features

The third author of this manuscript (DS) and a team of 4 EAs developed Harbor, which comprises 4 main modules. First, peers enter information about their EA friend’s substance use to get feedback on the seriousness of their friend’s problem. This module adapts the principles of brief motivational interventions by providing normative feedback, comparing an individual’s use with well-established norms. Such interventions are efficacious at reducing substance use by correcting misperceived norms about substance use prevalence [23]. This novel feature of Harbor delivers normative feedback to peers with hopes of engaging them in helping processes by raising awareness about the gravity of their friend’s substance use. This module may be useful for peers who are ambivalent about helping a friend with substance use problems. The second module teaches peers the principles of CRAFT through a series of mock text dialogues demonstrating confrontational, enabling, and supportive (ie, per the CRAFT intervention) responses to their EA friend’s retelling of a substance use–related incident (Multimedia Appendices 1-3). This study focused primarily on this feature of Harbor. Third, Harbor contains a module designed to discuss the idea of arranging competing prosocial activities at times when the EA friend would normally use substances. Finally, Harbor concludes with a module focused on training EA peers on how to have a discussion with their EA friends about seeking treatment.

### The Market Need for Harbor

A recent meta-analysis found that *only about 20% (*n=10) of studies with EA samples from noncollege settings used technology-assisted interventions [24]. Furthermore, most technology-assisted interventions included were preventative in nature and mostly targeted alcohol use. The Harbor app addresses these limitations. First, it galvanizes EA peers to act as first responders for their close friends who demonstrate risky substance use behaviors. Second, it teaches EA peers’ supportive skills to address multiple forms of substance use (ie, alcohol and cannabis use). In addition, a systematic review found that technology-assisted interventions worked equally well compared with the more traditional interventions for EAs in noncollege settings [24]. This finding suggests that the Harbor app may have expanded utility beyond traditional college settings where most research with EA samples occurs.

In examining the market for mobile apps related to Harbor, we found a dearth of programs designed to address the needs of concerned EAs with close friends who use substances. Most apps on the market target individuals attempting to ameliorate their own substance use issues and use common intervention strategies such as daily affirmations, drink tracking, and networking. Apps such as *Blood Alcohol Tracker*, *AlcoDroid*, *Substance Abuse and Addiction Assessments*, *Cravings Manager*, and *12 Steps AA Companion* currently exist for Apple, Android, and Blackberry users [25]. Each app has a unique self-reporting function and interfaces primarily with the client. Unlike the Harbor app, these apps do not involve another person to assist with changing behaviors. Although effective in reducing personal substance use or connecting individuals who may have substance use issues, these and other programs do not specifically focus on EAs. Furthermore, rather than focusing solely on recovery-oriented support (eg, Al-Anon Speaker Tapes and Al-Anon Audio Companion), a program such as Harbor could provide harm reduction alternatives to EA peers with concerns about a friend’s substance use along the full severity spectrum.

### Participant Recruitment

The authors obtained institutional review board approval before implementing the study procedures and informed consent from participants before their engagement with the app. Researchers conducted participant recruitment via Amazon’s Mechanical Turk (MTurk) program from April 19, 2016, to September 2, 2016. MTurk is an online pool of workers who complete surveys and similar tasks for remuneration. In addition, surveys completed by MTurk workers have yielded reliable data [26], and online MTurk studies have successfully replicated findings originally drawn from in-person studies [27].

Despite the promise of MTurk for data collection, additional safeguards are necessary to enhance data quality. First, we used qualification screening to confirm participants’ eligibility. After completing this qualification test and if eligible to participate in the study, participants completed the full survey. Participants were between the ages of 18 and 29 years and reported having consumed at least one alcoholic beverage in the past year. We eliminated survey responses completed too quickly (<5 min) and those missing validity check questions from the participant pool [27,28]. Finally, it is possible, although extremely unlikely, that one person would have multiple accounts and complete the full survey multiple times. However, Amazon’s requirement of providing a valid social security number during account setup should prevent one person from registering multiple times.

### Measures

#### Intervention Script Ratings

Participants read short text conversations used to teach the principles of enabling substance use, confronting another’s substance use, or encouraging change in their friend’s substance use via nonjudgmental responses that also encouraged substance-free activities (ie, supportive behaviors). Using 5-point Likert scales, participants then rated these scripts on how enabling (1=extremely discouraging use to 5=extremely enabling use), confrontational (1=extremely nonconfrontational to 5=extremely confrontational), or supportive (1=extremely unsupportive to 5=extremely supportive) they were. Furthermore, the authors prompted participants to respond with how accurately the scripts depict real-life conversations between EAs (1=extremely unrealistic to 5=extremely realistic) and how useful the scripts would be in teaching them how to support their friend’s sobriety (1=not helpful at all and 5=extremely helpful). Multimedia Appendices 1-3 show examples of confrontational, enabling, and supportive scripts respectively, as well as script rating items for each. Finally, respondents rated how likely they would be to use this mobile phone app or others like it based on various referral sources (1=completely unlikely and 5=completely likely).

#### Self-Reported Interactions With Friends

The authors asked eligible participants, “Do you currently have any friends for whom you have concerns about their substance use?” Those who replied affirmatively completed the Significant Other Behavior Questionnaire (SBQ), a valid and reliable (α=.85) measure of the significant other’s responses to an identified individual’s substance use [29-31]. Authors amended the wording of individual items of the SBQ to achieve greater relevance for EAs, close friend dyads (eg, “friend” replaced “significant other”). In addition, the adapted version of the SBQ included 1 revised item to eliminate the assumption that all peers live together (eg, “Do you refuse to be around your friend when they are drinking or using?” versus “Do you refuse to be home when they are drinking?”) and used “drinking and drug use” instead of “alcohol use” to account for multiple substances [20].

The amended SBQ contains 5-point Likert scale items (1=completely unlikely and 5=completely likely) clustered within 4 subscales: *support sobriety (SS)*, *support substance use (SSU; ie, enabling)*, *punish substance use (PSU; ie, confronting)*, and *withdrawal from friend (WFF)*. The SS subscale (8 items, range 8-40) measures the frequency with which a peer uses positive supportive strategies with their friend to encourage their friend to remain sober (eg*,* “Do you spend more time with your friend when they are not drinking or using drugs?”)*.* The SSU subscale (6 items, range 6-30) measures the frequency with which a peer has encouraged their friend to use substances (eg*,* “Do you bring alcoholic beverages/drugs to your friend?”)*.* The PSU subscale (5 items, range 5-25) measures the frequency with which a peer uses coercive or otherwise negative strategies to stop their friend from using substances (eg, “Do you have arguments regarding drinking or drug use when your friend is using?”). The WFF subscale (5 items, range 5-25) measures the frequency with which a peer attempts to withdraw from their friend while they are using substances (eg, “Do you refuse to be around your friend when they are drinking or getting high?”).

#### Substance Use

To measure participants’ personal substance use, we used the Alcohol Use Disorder Identification Test (AUDIT), a valid and reliable (α=.88) measure of risky alcohol use [32-34]. Along with the AUDIT, we measured peers’ related substance use problems with the Substance Problem Scale (SPS, past month version), a 16-item scale from the larger Global Assessment of Individual Needs-I. Each item consists of a 4-point Likert scale indicating the last time an individual experienced a particular substance use–related issue (0=never, 1=more than a year ago, 2=past year, and 3=past month). We recoded responses to SPS items to reflect the past year problems categorically (0=not past year, 1=past year/month). The SPS contains items consistent with the Diagnostic and Statistical Manual of Mental Disorders criteria for substance use disorders and some additional items related to risky substance use behaviors (eg, hiding use) [35]. Furthermore, SPS has been used in various EA studies [36-38], demonstrated good reliability (α=.85) in predicting substance use disorders with a nationally representative sample of EAs [39], cross-validated with independently made psychiatric diagnoses [40], and is correlated with perceived EA status [38].

### Data Analysis

The authors conducted analyses using SPSS 25.0 [41], first checking distributions for normality and assessing missing data, which were minimal. Nevertheless, analysts used multiple imputations through which statistical software imputed missing variables 5 times, analyzed each of the completed data sets, and pooled the results of each analysis into a final data set. The final results did not significantly differ from the original, unadjusted analyses. The authors present descriptive analyses of demographic data, individual SBQ items and SBQ subscales, and full-scale scores from the SPS and AUDIT. Furthermore, the authors estimated the potential market size of individuals that may benefit from using the mobile app. In addition, analysts calculated Pearson product-moment correlations between the SBQ, its subscales, and intervention script ratings and intercorrelations between SBQ subscales. Finally, analysts calculated Pearson correlations between these intervention script ratings and personal measures of risky drinking behaviors (ie, AUDIT) as well as between the SBQ subscale and AUDIT scores.

## Results

### Demographics

Table 1 illustrates the demographic characteristics of our sample. On average, the full EA sample included participants who were 25.4 years old; among them, 58.1% (266/458) identified as male and 72.7% (333/458) identified as White. We note that the proportion of the sample working 35 or more hours per week (248/458, 54.1%) is lower than that reported in many national, multisite clinical trials [5]. Participants reported a median, past year income of roughly US $23,000, and a mean MacArthur Scale of Subjective Social Status [42] of 4.29. Our sample had a mean SPS (past year) score of 2.56 (range 0-16) and a mean AUDIT score of 6.38 (range 0-36). In both cases, higher scores represent greater issues pertaining to substance use.

Table 2 contains the SBQ individual item results as well as subscale scores.

**Table 1:** Participant demographic characteristics (N=458).

Characteristics		Value
Age (years), mean (SD)		25.39 (2.84)
Age at first substance use (years), mean (SD)		17.18 (2.60)
Male, n (%)		270 (59.0)
**Race/ethnicity, n (%)**		
	White	333 (72.7)
	Asian	47 (10.3)
	African American	32 (7.0)
	Hispanic/Chicano/Latino	31 (6.8)
	American Indian/Alaskan Native	5 (1.1)
	Native Hawaiian/Pacific Islander	1 (0.2)
	Other	9 (2.0)
**Education/occupation, n (%)**		
	Any postsecondary education	270 (59.0)
	Employed full time	248 (54.1)
	Employed part time	117 (25.5)
	Unemployed	93 (20.3)
	Unenrolled and unemployed	40 (8.7)
**Current enrollment status, n (%)**		
	Not enrolled	188 (41.0)
	Four-year college/university	186 (40.6)
	Two-year community/junior college	55 (12.0)
	High school	12 (2.6)
	Technical school	10 (2.2)
	Job training program	5 (1.1)
	GED^a^ classes	2 (0.4)
**Relationship status, n (%)**		
	Single	215 (46.9)
	Married	90 (19.6)
	Divorced	4 (0.9)
	In a serious relationship	149 (32.5)
**Substance use, mean (SD)**		
	SPS^b^ score (past year)	2.56 (4.23)
	AUDIT^c^ score	6.38 (5.69)

^a^GED: general equivalency diploma.

^b^SPS: Substance Problem Scale.

^c^AUDIT: Alcohol Use Disorder Identification Test.

**Table 2:** Peer Significant Other Behavior Questionnaire subscale responses to friend’s substance use (n=208).

SBQ^a^ item and subscale (1=highly unlikely, 5=highly likely)		Mean (SD)
**Do you…**		
	stop your friend’s drinking and drug use by getting angry (PSU^b^)	2.07 (0.78)
	have arguments regarding drinking and drug use when your friend is using (PSU)	2.62 (0.85)
	show how unhappy you are when your friend is drinking or using drugs (PSU)	3.49 (0.82)
	scare your friend from drinking or using drugs by warning (PSU)	2.29 (0.83)
	tell your friend about things they did when drunk or high (PSU)	3.87 (0.78)
	spend more time with your friend when they are not drinking or using (SS^c^)	3.73 (0.83)
	do things your friend likes when your friend is not drinking or using (SS)	3.96 (0.67)
	give your friend compliments when they are not drinking or using (SS)	3.79 (0.77)
	enjoy your friend’s company when they are not drinking or using (SS)	4.21 (0.67)
	do things for your friend when they are not drinking or using (SS)	3.93 (0.67)
	support your friend when he or she is having trouble staying sober (SS)	4.06 (0.68)
	arrange nondrinking or using social outings (SS)	3.79 (0.76)
	when your friend is not drinking or using, do you enjoy each other (SS)	4.38 (0.52)
	when your friend is drinking or using, do you join him/her (SSU^d^)	2.62 (0.87)
	bring alcoholic beverages or drugs to your friend's house (SSU)	1.94 (0.77)
	buy alcohol for your friend when you are at bars, etc (SSU)	1.97 (0.77)
	do things your friend likes when your friend is drinking or using (SSU)	2.67 (0.84)
	tell your friend it is ok to have “just two or three” (SSU)	2.53 (0.89)
	tell your friend they are fun to be with when they are drinking or using (SSU)	1.97 (0.71)
	leave social situations when your fiend is drinking or using (WFF^e^)	3.02 (0.83)
	refuse to be around your friend when they are drinking or using (WFF)	2.91 (0.89)
	keep out of your friend’s way when he or she is drunk or high (WFF)	3.16 (0.82)
	make other plans to go out alone or with others when your friend has been drinking or using (WFF)	3.51 (0.81)
	refuse to take care of your friend when they are drunk or high (WFF)	2.26 (0.82)
**Subscale**		
	Punish substance use	2.87 (0.55)
	Support sobriety	3.98 (0.51)
	Support substance use	2.28 (0.60)
	Withdraw from friend	2.98 (0.67)

^a^SBQ: Significant Other Behavior Questionnaire.

^b^PSU: punish substance use.

^c^SS: support sobriety.

^d^SSU: support substance use.

^e^WFF: withdraw from friendship.

### Hypothesis 1: Market Size/Acceptability

Approximately half of the participants (208/458, 45.4%) indicated that they had a friend experiencing problems with alcohol or drugs. A majority of the sample (334/458, 72.9%) reported they would be *somewhat* *likely* or *completely likely* to use the mobile app, although the likelihood of use varied depending on the referral source. Fewer participants (222/458, 48.5%) reported that they would be at least *somewhat likely* to search app stores on their own for such an app. However, if their close friend or someone directly associated with them (eg*,* their therapist, another close friend, a family member) asked them to use the app, 81.2% *(*372/458) of participants reported they would be at least *somewhat likely* to use it.

### Hypothesis 2: Participant Intervention Script Ratings

Overall, participants rated the intervention scripts favorably in terms of linguistic/cultural accuracy and overall script utility. Participants comprehensively rated the scripts as *slightly realistic* or better overall (mean 3.92, SD 0.48). Regarding utility, an overwhelming majority of peers (418/458, 91.3%) rated the scripts as at least slightly helpful in assisting them to support their friends who use substances. Participants identified scripts specifically designed to be enabling (mean 4.42, SD 0.49) and confrontational (mean 4.28, SD 0.50) as such. However, participants rated supportive scripts more neutrally (mean 2.96, SD 0.62).

### Hypothesis 3: Participant Intervention Script Ratings and Self-Reported Interactions With Friends

Table 3 displays correlations between SBQ subscales (ie, self-reported actual behaviors) and confrontational, enabling, and supportive script ratings. Overall, the sample had higher scores for both enable drinking and confront drinking subscales relative to prior studies [43,44]. As hypothesized, there was a significant negative correlation (*r*_206_=−0.236, *P*=.001) between enabling script ratings and scores on the SSU subscale of the SBQ, indicating that those who reported more enabling behaviors in real life rated the intervention script dialogue as less enabling. Similarly, there was a significant positive correlation between enabling script ratings and scores on the SS subscale of the SBQ (*r*_206_=0.375, *P*<.001).

As hypothesized, a positive correlation (*r*=0.170, *P*=.01) existed between supporting sobriety and confrontational script ratings, indicating that EAs who already support their friend’s sobriety were more successful in identifying confrontational language. Conversely, correlations between supportive script ratings and the SS subscale were nonsignificant. Furthermore, nonsignificant correlations emerged between confrontational script ratings and the PSU subscale of the SBQ.

Participants rated to what degree they perceived these three scripts as supportive, enabling, or confrontational.

Table 4 displays intercorrelations between the SBQ subscales. Most were in the expected directions based on prior theory and research. For instance, participant scores on the SS subscale correlated negatively with scores on the SSU subscale (*r*=−0.207, *P*=.003).

**Table 3:** Script ratings and Significant Other Behavior Questionnaire subscale correlations.

Script type		PSU^a^	SS^b^	SSU^c^	WFF^d^
**Supportive script**					
	Correlation	0.054	0.086	*0.157* ^e^	0.031
	*P* value (two-tailed)	.44	.22	.02	.66
	Participants, n	208	208	208	208
**Enabling script**					
	Correlation	*0.154*	*0.375*	−*0.236*	*0.274*
	*P* value (two-tailed)	.03	<.001	.001	<.001
	Participants, n	208	458	458	458
**Confrontational script**					
	Correlation	0.008	*0.170*	−0.071	0.060
	*P* value (two-tailed)	.91	.01	.31	.39
	Participants, n	208	208	208	208

^a^PSU: punish substance use.

^b^SS: support sobriety.

^c^SSU: support substance use.

^d^WFF: withdraw from friendship.

^e^Italicized data represent findings significant at a level of <.05.

**Table 4:** Significant Other Behavior Questionnaire subscale intercorrelations.

SBQ subscale		PSU^a^	SS^b^	SSU^c^	WFF^d^
**PSU**					
	Correlation	1	*0.406* ^e^	0.004	*0.287*
	*P* value (two-tailed)	—^f^	<.001	.94	<.001
	Participants, n	208	208	208	208
**SS**					
	Correlation	*0.406*	1	*−0.207*	*0.452*
	*P* value (two-tailed)	<.001	—	<.001	<.001
	Participants, n	208	208	208	208
**SSU**					
	Correlation	0.004	*−0.207*	1	*−0.389*
	*P* value (two-tailed)	.94	.003	—	<.001
	Participants, n	208	208	208	208
**WWF**					
	Correlation	*0.287*	*0.452*	*−0.389*	1
	*P* value (two-tailed)	<.001	<.001	<.001	—
	Participants, n	208	208	208	208

^a^PSU: punish substance use.

^b^SS: support sobriety.

^c^SSU: support substance use.

^d^WFF: withdraw from friendship.

^e^*Italicized* data represent findings significant at a level of <.05.

^f^Data not applicable (1:1 correlations).

Individuals who scored higher on the SS subscale of the SBQ also rated the scripts as more accurate overall in terms of the language used being representative of how EAs may text or converse with one another in more natural contexts (*r*=0.136, *P*=.001). Conversely, EAs who scored higher on the SS subscale of the SBQ rated the scripts as less useful overall (Table 5; *r*=−0.144, *P*=.04). Table 5 presents the correlations for the SBQ subscales with mean script accuracy and utility ratings across all scripts. Finally, aligned with a priori hypotheses, enabling script ratings were negatively correlated with AUDIT scores (*r*=−0.139, *P*=.003). In other words, participants with higher AUDIT scores rated enabling scripts lower (ie, lower perceived enabling).

Table 6 displays the correlations between the AUDIT scores and intervention script ratings.

This suggests that those with higher AUDIT scores may perceive their own substance use and attitudes toward their friends’ substance use as nonenabling and similarly be an example of a subgroup that may benefit from using the Harbor app.

**Table 5:** Significant Other Behavior Questionnaire subscale and overall script accuracy and utility ratings.

SBQ^a^ subscale		Script accuracy	Script utility
**PSU^b^**			
	Pearson correlation	0.073	0.010
	*P* value (two-tailed)	.29	.88
	Participants, n	208	208
**SS^c^**			
	Pearson correlation	*0.204* ^d^	−*0.144*
	*P* value (two-tailed)	.003	.04
	Participants, n	208	208
**SSU^e^**			
	Pearson correlation	−0.067	−0.010
	*P* value (two-tailed)	.34	.89
	Participants, n	208	208
**WFF^f^**			
	Pearson correlation	0.021	0.035
	*P* value (two-tailed)	.77	.61
	Participants, n	208	208

^a^SBQ: Significant Other Behavior Questionnaire.

^b^PSU: punish substance use.

^c^SS: support sobriety.

^d^Italicized data represent findings significant at a level of <.05.

^e^SSU: support substance use.

^f^WFF: withdraw from friendship.

**Table 6:** Alcohol Use Disorder Identification Test score and script rating correlations.

Substance use measure		Confrontational script	Enabling script	Supportive script
**AUDIT^a^ score**				
	Pearson correlation	−0.020	−*0.139*^b^	*0.107*
	*P* value (two-tailed)	.66	.003	.02
	Participants, n	458	458	458

^a^AUDIT: Alcohol Use Disorder Identification Test.

^b^*Italicized* data represent findings significant at a level of <.05.

## Discussion

### Principal Findings

This study investigated 3 research questions: What is the potential market size of EAs who may use an app such as Harbor? What do potential Harbor users think of our intervention scripts? How do script ratings associate with self-reported participant behaviors toward existing friends’ substance use problems? Systematic reviews on the functionality, feasibility, and acceptability of smartphone apps for EAs reveal a need for more rigorous evaluations of the perceived utility of and demand for apps [45]. From 2015 to 2019, smartphone ownership among EAs increased from 85% to 96% [46,47]. Considering this, reaching EAs with substance use problems through technology is an attractive option.

Almost half of our sample (208/458, 45.4%) reported having a friend for whom they currently had concerns about their substance use, and 81.4% (373/458) of our survey respondents indicated they would be likely to download and use the Harbor app depending upon the referral source. This willingness to use such a program suggests a strong demand for a mobile app such as Harbor, as many EAs may have friends for whom they have concerns regarding substance use but lack the training and/or confidence to broach such issues with them. In addition, many participants in our sample were able to identify the supportive, confrontational, or enabling language within the scripts. Overall, these findings indicate that the app is acceptable to participants, and interpretations may vary depending on the user.

Although participants’ script utility ratings indicated some ambivalence about how helpful the app’s scripts would be, the pattern of correlations makes a theoretical sense, that is, many of the correlations between the SBQ and intervention script ratings indicate that participants would have room for growth in communications with their EA peers affected with substance use. For instance, enabling script ratings correlated negatively with self-reported enabling behaviors in real life. This suggests that EAs’ peers who self-reported enabling behaviors rate enabling scripts lower. This may indicate that decreasing enabling is a viable change target among such peers. Such findings may also support the construct validity of the single item used to measure the enabling script.

Conversely, those who already understand the concept of the role of enabling behavior in contributing to their peers’ substance use may not benefit from the training module. The positive correlations between the SS subscale and both the enabling and confrontation script ratings underscore this point. Those who already use science-based communication and support their peers’ sobriety likely recognize enabling and confrontational behaviors when they see them. For such individuals, the app may not teach new communication skills but rather affirm the skills they already have. This may help explain the negative correlation between supporting sobriety scores and script utility ratings (*r*_206_=−0.097, *P*=.04). Although they rated the scripts as linguistically accurate, individuals who are already engaging in supportive behaviors seem to perceive their lack of personal need for such an app. The negative correlation could also indicate that those low on supportive behaviors rated the communication skills module as more useful, affirming its need among those for whom the content appeared novel.

Furthermore, these associations generally support the validity of these constructs among EAs. There are few studies on close friend communication patterns about substance use in the context of one peer having elevated use. To our knowledge, this is only the second study of SBQ measures involving EAs, and both support the notion that enabling, confrontation, and supporting sobriety are relevant concepts for peers with friends who misuse substances. It is encouraging that these associations support the theory-based (ie, CRAFT) intervention. Future research on Harbor could test whether confrontation, enabling, and support change over time for individuals who use the app.

Finally, it may be that the lower-than-expected ratings on the utility of the app were due to only showing select content of the app, that is, participants in this study did not see the entire intervention. Consequently, although they received a brief orientation to the purpose of the app before the presentation of the mock scripts, a lack of context about the rationale for speaking with friends about substance use in a specific way may have affected ratings. This study demonstrates some initial support for the acceptability and need for the intervention. However, future research on satisfaction with the Harbor app should come from actual users of the mobile app exposed to all content.

### Limitations

Researchers and practitioners should examine the insights gained from this study in conjunction with the study’s limitations. First, although the sample size is larger than or comparable to other feasibility studies, including technology-assisted interventions [48-51], it still may not be generalizable to the general EA population. It is also unclear whether peer reports of willingness to use the mobile app would translate to an increase in their supportive behaviors, and ultimately, a reduction in their close friend’s substance use. Another potential limitation is the use of MTurk and self-reported data; however, according to prior research [52,53], both online survey data collection and self-reported data have demonstrated validity and reliability as research methods. To strengthen the reliability of the data gained from this study, researchers embedded several validity check items within the online survey. Furthermore, it should be noted that participants in this study did not download and interact with the app; they merely saw static screenshots and rated some of the scripts from 1 of 4 modules. Furthermore, additional engagement with the CSOs throughout intervention testing could build upon the CBPR framework used to guide this initial study. At the time of writing this paper, we have completed qualitative interviews with participants in the face-to-face version of the intervention. We did not verify survey findings with respondents, which would have enhanced participant involvement.

### Future Studies

Mobile apps can make behavioral health care more accessible, interactive, and efficient [15]. In general, clients find digital interventions easy to operate and useful, and rates of prolonged use are generally high [54]. This study demonstrated the acceptability and feasibility of Harbor, a mobile app designed to teach EAs who have friends with substance use problems ways to intervene with their friends’ substance use. Substance use in EAs is a major public health problem [55], and Harbor may be useful in extending both the reach and efficacy of treatments targeting this population. The findings also demonstrated the construct validity of enabling, confronting, and supporting behaviors among peers of EAs who may be struggling with substance use problems. Future research on Harbor can test its efficacy in improving EAs’ substance use treatment outcomes by altering these peer behaviors.

## Appendix

Multimedia Appendix 1 Screenshot of example confrontational script and associated questionnaire items.

Multimedia Appendix 2 Screenshot of example enabling script and associated response item.

Multimedia Appendix 3 Screenshot of supportive script and associated response item.

## Abbreviations

AUDIT: Alcohol Use Disorder Identification Test
CBPR: community-based participatory research
CRAFT: Community Reinforcement Approach With Family Training
CSOs: concerned significant others
EA: emerging adult
MTurk: Amazon’s Mechanical Turk
Peer-CRA: peer-enhanced community reinforcement approach
PSU: punish substance use
SBQ: Significant Other Behavior Questionnaire
SPS: Substance Problem Scale
SS: support sobriety
SSU: support substance use
