# Supplementary figures and images for: Usability and Acceptability of a Mobile App to Help Emerging Adults Address their Friends' Substance Use (Harbor): Quantitative Study
Source: J Med Internet Res. 2020 Nov 5;22(11):e16632. doi: 10.2196/16632 (PMC7677020; doi:10.2196/16632)

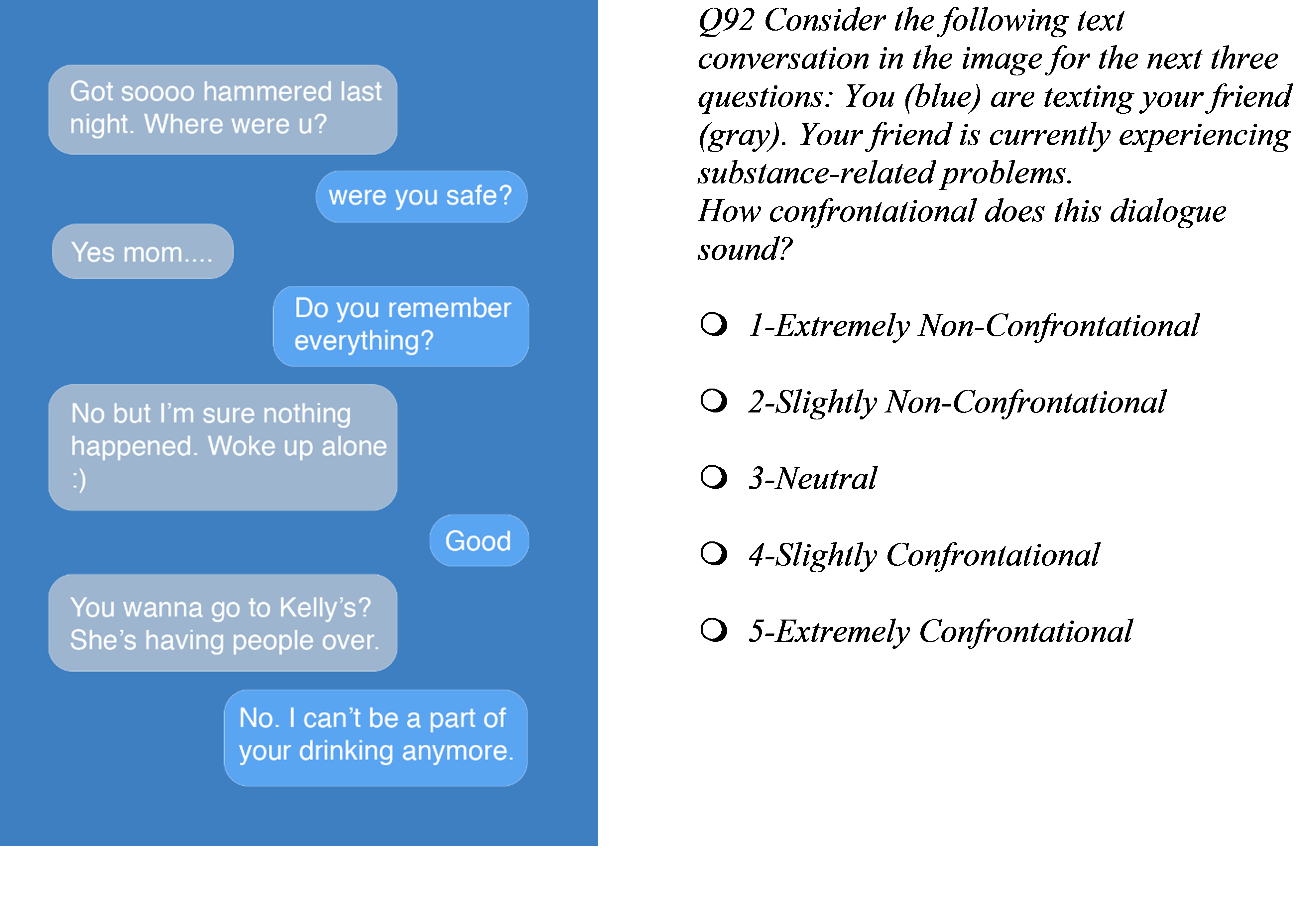

Supplement: Multimedia Appendix 1 [file jmir_v22i11e16632_app1.png]

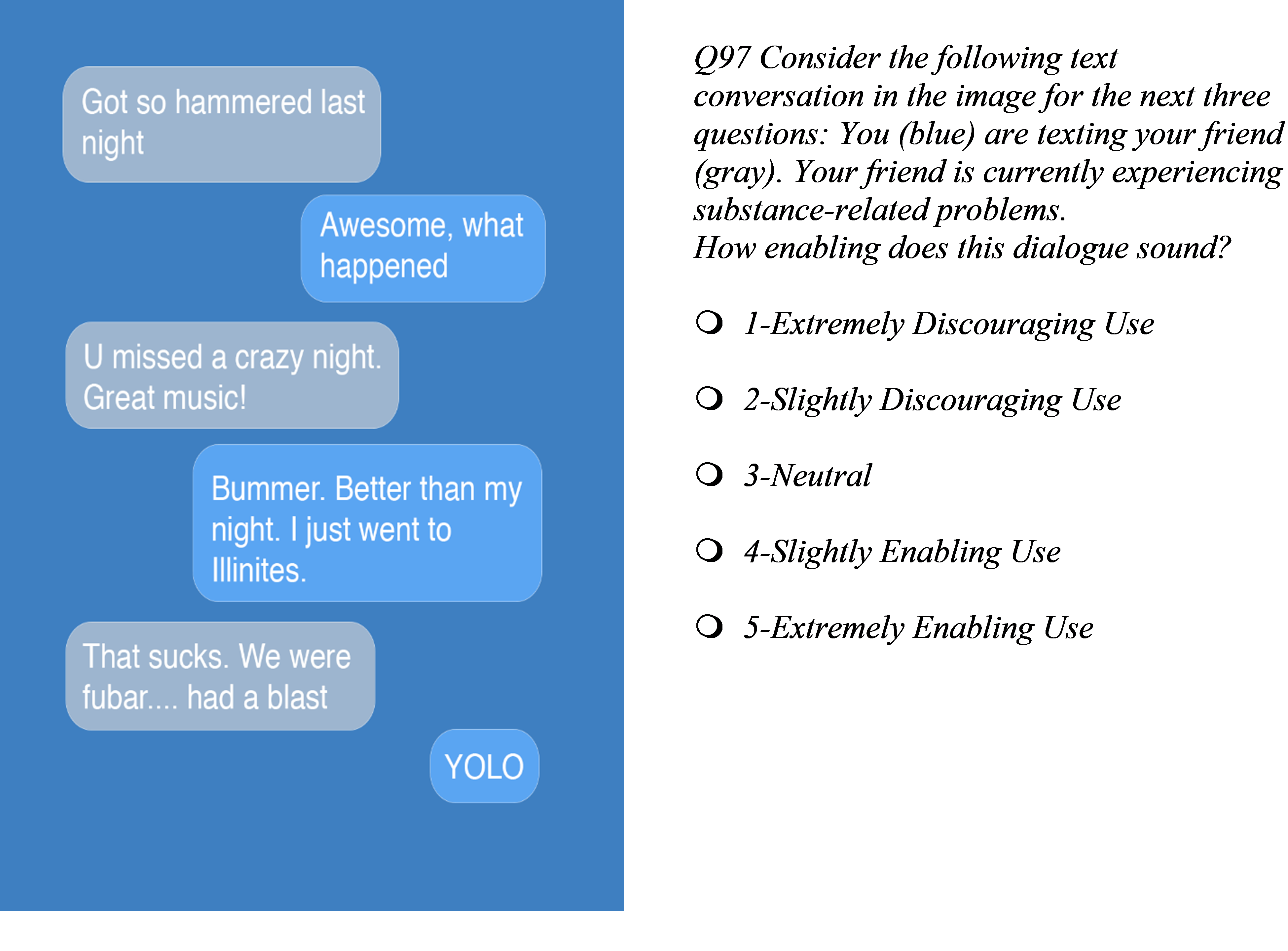

Supplement: Multimedia Appendix 2 [file jmir_v22i11e16632_app2.png]

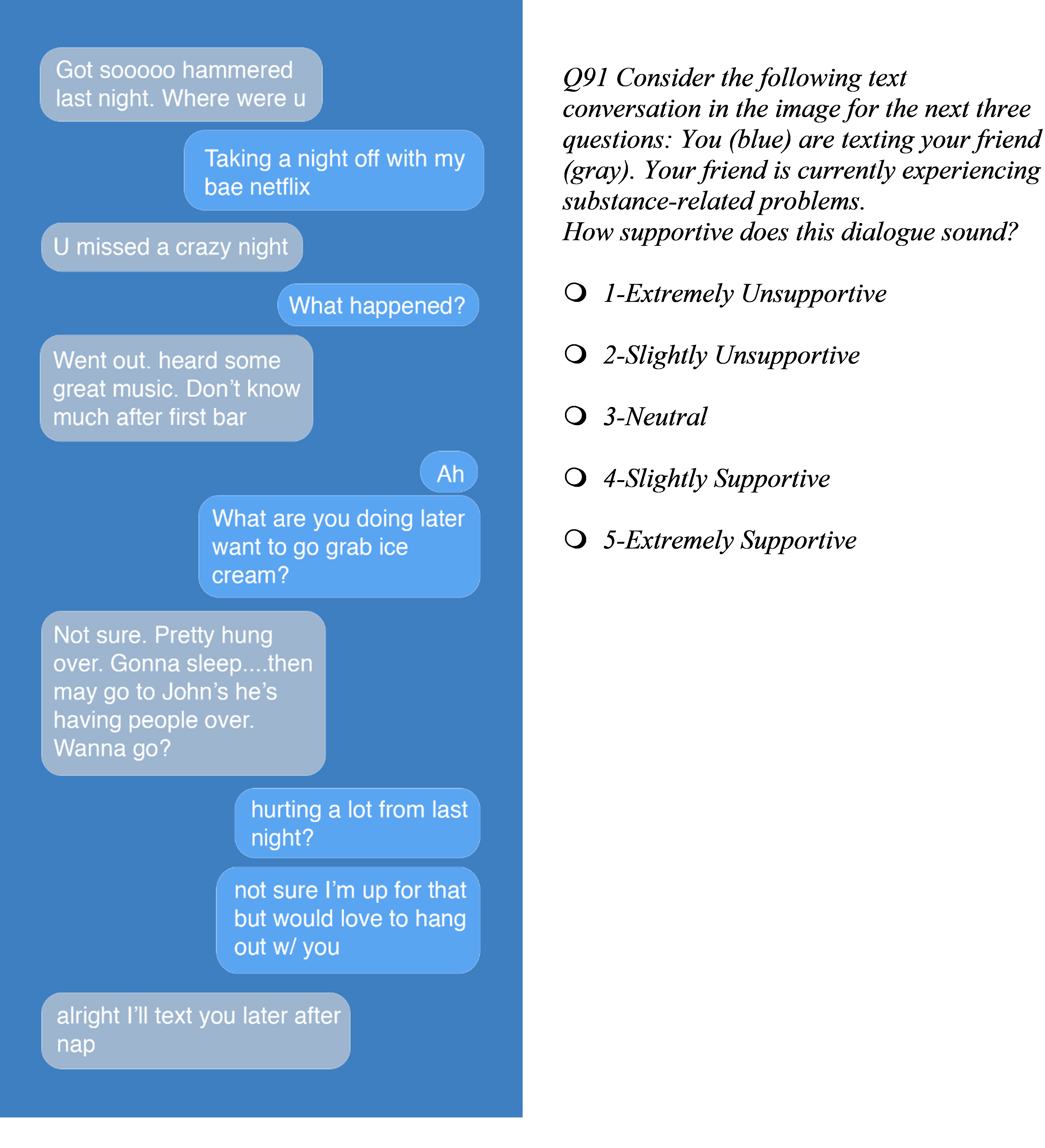

Supplement: Multimedia Appendix 3 [file jmir_v22i11e16632_app3.png]
